# 4,4′-Bipyridinium bis­(μ-4-oxo-1,4-dihydropyridine-2,6-dicarboxyl­ato)bis­[aqua­hydroxido­anti­monate(III)] dihydrate

**DOI:** 10.1107/S1600536810010743

**Published:** 2010-03-31

**Authors:** Janet Soleimannejad, Jafar Attar Gharamaleki, Hossein Aghabozorg, Yaghoub Mohammadzadeh Azar Golenji

**Affiliations:** a Department of Chemistry, Ilam University, Ilam, Iran; b Young Researchers Club, Islamic Azad University, North Tehran Branch, Tehran, Iran; c Faculty of Chemistry, Islamic Azad University, North Tehran Branch, Tehran, Iran

## Abstract

The title compound, (C_10_H_10_N_2_)[Sb_2_(C_7_H_2_NO_5_)_2_(OH)_2_(H_2_O)_2_]·2H_2_O, consists of a binuclear anion, a diprotonated 4,4′-bipyridinium cation and two uncoordinated water mol­ecules. Each Sb^III^ atom is six-coordinated by one chelating 4-oxidopyridine-2,6-dicarboxyl­ate ligand, one water mol­ecule, one OH group and one bridging O atom from a neighboring carboxyl­ate group in a distorted penta­gonal-pyramidal geometry, with the OH group at the apical position. The two pyridine rings in the bipyridinium cation are twisted with respect to each other, making a dihedral angle of 22.7 (1)°. The cations are connected to the anions by N—H⋯O hydrogen bonds, forming a chain. The coordinated water mol­ecules form hydrogen bonds with the oxido O atoms of the anion, building a two-dimensional sheet, which is further connected into a three-dimensional structure by O—H⋯O and C—H⋯O hydrogen bonds and C=O⋯π inter­actions [O⋯centroid distances = 3.1785 (17), 3.4737 (19) and 3.5685 (19) Å].

## Related literature

For the use of 4,4′-bipyridine in the construction of supra­molecular architectures, see: Jia *et al.* (2009 ▶); Meng *et al.* (2009 ▶); Zhang *et al.* (2009 ▶). For binuclear complexes of Sb^III^/Sb^V^ with pyridine-2,6-dicarboxylic acid, see: Aghabozorg *et al.* (2005 ▶); Soleimannejad *et al.* (2008 ▶). For proton transfer compounds and their metal complexes, see: Aghabozorg *et al.* (2008 ▶). For environmental studies of anti­mony, see: Filella *et al.* (2002 ▶).
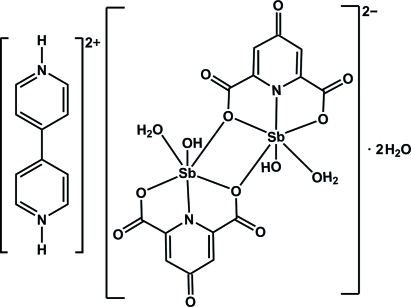


## Experimental

### 

#### Crystal data

(C_10_H_10_N_2_)[Sb_2_(C_7_H_2_NO_5_)_2_(OH)_2_(H_2_O)_2_]·2H_2_O
*M* *_r_* = 867.97
Triclinic, 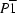

*a* = 7.7774 (9) Å
*b* = 10.2465 (12) Å
*c* = 17.773 (2) Å
α = 80.255 (5)°
β = 81.760 (5)°
γ = 82.547 (5)°
*V* = 1373.4 (3) Å^3^
*Z* = 2
Mo *K*α radiation
μ = 2.06 mm^−1^
*T* = 150 K
0.32 × 0.32 × 0.13 mm

#### Data collection

Bruker SMART 1000 CCD diffractometer
Absorption correction: multi-scan (*SADABS*; Bruker, 2001 ▶) *T*_min_ = 0.559, *T*_max_ = 0.776
29846 measured reflections
6323 independent reflections
5762 reflections with *I* > 2σ(*I*)
*R*_int_ = 0.027

#### Refinement

*R*[*F* ^2^ > 2σ(*F* ^2^)] = 0.021
*wR*(*F* ^2^) = 0.054
*S* = 1.05
6323 reflections
415 parameters
H-atom parameters constrained
Δρ_max_ = 1.03 e Å^−3^
Δρ_min_ = −0.61 e Å^−3^

### 

Data collection: *SMART* (Bruker, 2007 ▶); cell refinement: *SAINT* (Bruker, 2007 ▶); data reduction: *SAINT*; program(s) used to solve structure: *SHELXS97* (Sheldrick, 2008 ▶); program(s) used to refine structure: *SHELXL97* (Sheldrick, 2008 ▶); molecular graphics: *SHELXTL* (Sheldrick, 2008 ▶); software used to prepare material for publication: *SHELXTL*.

## Supplementary Material

Crystal structure: contains datablocks I, global. DOI: 10.1107/S1600536810010743/hy2287sup1.cif

Structure factors: contains datablocks I. DOI: 10.1107/S1600536810010743/hy2287Isup2.hkl

Additional supplementary materials:  crystallographic information; 3D view; checkCIF report

## Figures and Tables

**Table 1:** Hydrogen-bond geometry (Å, °)

*D*—H⋯*A*	*D*—H	H⋯*A*	*D*⋯*A*	*D*—H⋯*A*
O1*W*—H1*A*⋯O2^i^	0.85	1.88	2.725 (2)	177
O1*W*—H1*B*⋯O11^ii^	0.85	1.87	2.704 (2)	167
O2*W*—H2*A*⋯O7^iii^	0.86	1.85	2.703 (2)	171
O2*W*—H2*B*⋯O16^iv^	0.85	1.86	2.669 (2)	157
O11—H11*A*⋯O2*W*	0.85	1.90	2.688 (2)	154
O16—H16*A*⋯O1*W*	0.85	1.88	2.684 (2)	155
O13—H13*A*⋯O9	0.85	1.83	2.672 (2)	173
O13—H13*B*⋯O3^v^	0.85	1.92	2.764 (2)	173
O15—H15*A*⋯O4	0.85	1.88	2.709 (2)	166
O15—H15*B*⋯O8^vi^	0.85	1.98	2.823 (2)	171
N3—H3*A*⋯O3^vii^	0.85	1.78	2.624 (2)	171
N4—H4*A*⋯O8^vi^	0.85	1.80	2.648 (2)	173
C3—H3⋯O1*W*^i^	0.93	2.60	3.460 (3)	154
C5—H5⋯O9^vi^	0.93	2.53	3.434 (3)	164
C12—H12⋯O4^v^	0.93	2.55	3.470 (3)	172
C15—H15⋯O6	0.93	2.59	3.415 (3)	149
C19—H19⋯O2*W*^iii^	0.93	2.60	3.484 (3)	160
C20—H20⋯O1*W*^viii^	0.93	2.25	3.172 (3)	172
C21—H21⋯O1^ix^	0.93	2.44	3.358 (3)	170
C22—H22⋯O2^ix^	0.93	2.45	3.107 (3)	128
C24—H24⋯O16^x^	0.93	2.57	3.261 (3)	131

Symmetry codes: (i) 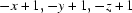; (ii) 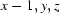; (iii) 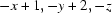; (iv) 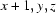; (v) 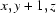; (vi) 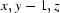; (vii) 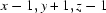; (viii) 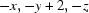; (ix) 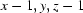; (x) 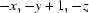.

**Fig. 1.**
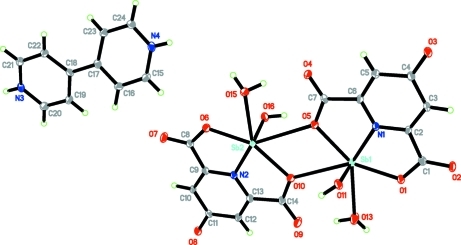
Molecular structure of the title compound. Displacement ellipsoids are shown at the 50% probability level. Two uncoordinated water molecules are omitted for clarity.

**Fig. 2.**
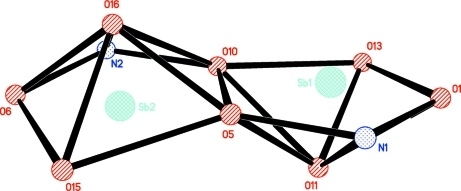
Distorted pentagonal pyramidal geometries around the SbIII atoms in the anionic complex.

**Fig. 3.**
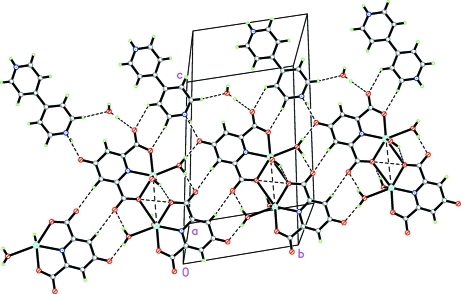
Hydrogen bonding (dashed lines) between 4,4'-bipyridinium cations, [Sb(hypydc)(OH)(H2O)]22- anions and uncoordinated water molecules.

**Fig. 4.**
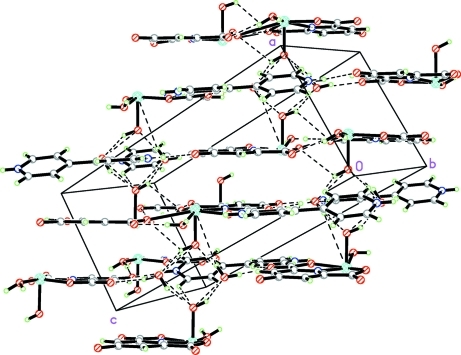
Crystal packing of the title compound. Hydrogen bonds are shown as dashed lines.

## supplementary crystallographic information

### Comment

It is well known that 4,4'-bipyridine is an excellent candidate for the construction of three-dimensional network motifs. It can act as coordinating or bridging ligand or as proton acceptor agent (Jia *et al.*, 2009; Meng *et al.*, 2009; Zhang *et al.*, 2009). The binuclear mixed ligand Sb^III^/Sb^V^ compounds have been recently reported, in which pyridinedicarboxylic acid has been used as chelating ligand (Soleimannejad *et al.*, 2008). For more details regarding proton transfer compounds and their metal complexes see our review article (Aghabozorg *et al.*, 2008).

The title compound is composed of a binuclear [Sb(hypydc)(OH)(H_2_O)]_2_^2-^ (H_3_hypydc = 4-hydroxypyridine-2,6-dicarboxylic acid) anion, a diprotonated 4,4'-bipyridinium cation and two uncoordinated water molecules (Fig. 1). In the binuclear complex anion, each Sb^III^ atom is six-coordinated by one chelating (hypydc)^3-^ ligand through its one N and two O atoms, one O atom from a terminal OH group, one O atom from a water molecule and one O atom from a neighboring carboxylate group as bridging ligand in a distorted pentagonal pyramidal geometry (Fig. 2). The relatively weaker Sb1—O10 [2.7617 (14) Å] and Sb2—O5 [2.7959 (14) Å] interactions connect the two anionic units into a binuclear complex through sharing O atoms of the carboxylate groups. The OH group is located at the apical position [Sb1—O11 = 1.9300 (15) and Sb2—O16 = 1.9304 (14) Å] and the rest O_4_N set forms the basal plane. It is supposed that this OH group is formed during the partial hydrolysis of SbCl_3_ to SbOCl, which is further hydrolyzed to Sb(OH)_3_, because free Sb^III^ ion is stable in solution only at very high acidities (Filella *et al.*, 2002). The Sb1—N1 and Sb2—N2 bond distances are 2.197 (3) and 2.191 (3) Å, respectively, which are comparable with the bond distances of Sb^III^ binuclear complex with pyridine-2,6-dicarboxylic acid (Aghabozorg *et al.*, 2005). The Sb^III^ atoms deviate by 0.272 (5) and 0.249 (2) Å from the mean basal planes. The bond angles indicate that the lone-pairs on Sb^III^ atoms are stereochemically active and stand at the *trans* positions to the OH groups. In the 4,4'-bipyridinium cation, the two pyridine rings are twisted with respect to each other, making a dihedral angle of 22.7 (1)°, which indicates the flexibility of the central C—C bond.

The intramolecular hydrogen bonds O13—H13A···O9 and O15—H15A···O4 are present in the anionic complex (Table 1). The 4,4'-bipyridinium cations are hydrogen bonded to these anions by two distinct N3—H3A···O3^vii^ and N4—H4A···O8^vi^ hydrogen bonds [symmetry codes: (vi) *x*, *y*-1, *z*; (vii) *x*-1, *y*+1, *z*-1], forming one-dimensional chains. The coordinated water molecules act as donors with respect to oxido O atoms of the (hypydc)^3-^ ligands from neighboring chains, building a two-dimensional sheet (Fig. 3). These sheets are further connected into a three-dimensional structure by O—H···O and C—H···O hydrogen-bonding interactions involving coordinated terminal OH group as well as uncoordinated water molecules. A noticeable feature of the title compound is the presence of C═O···π interactions between C═O groups and pyridyl rings of 4,4'-bipyridinium cations. The O···π distances are 3.4737 (19) Å for C4═O3···*Cg*1 (1-*x*, 1-*y*, -*z*), 3.1785 (17) Å for C11═O8···*Cg*2 (-*x*, 2-*y*, -*z*) and 3.5685 (19) Å for C14═O9···*Cg*1 (-*x*, 2-*y*,-*z*) [*Cg*1 and *Cg*2 are the centroids of N3, C18—C22 ring and N4, C15, C16, C17, C23, C24 ring]. The crystal packing diagram of the title compound is shown in Fig. 4.

### Experimental

The title compound was prepared by the refluxing of 4,4'-bipyridine (312 mg, 2 mmol), 4-hydroxypyridine-2,6-dicarboxylic acid (183 mg, 1 mmol) and SbCl_3_ (228 mg, 1 mmol) in water (50 ml) in a 2:1:1 molar ratio for several hours. Colorless crystals were obtained by slow evaporation of the solvent at room temperature. The highest residual electron density was found 0.92 Å from O13 and the deepest hole 0.79 Å from Sb2.

### Refinement

C- and N-bound H atoms were positioned geometrically and treated as riding atoms, with C—H = 0.93 and N—H = 0.85 Å and with *U*_iso_(H) = 1.2*U*_eq_(C, N). H atoms on water molecules and hydroxy groups were observed on a difference Fourier map and refined as riding, with O—H = 0.85 Å and *U*_iso_(H) = 1.2*U*_eq_(O).

### Crystal data

**Table:**

(C_10_H_10_N_2_)[Sb_2_(C_7_H_2_NO_5_)_2_(OH)_2_(H_2_O)_2_]·2H_2_O	*Z* = 2
*M**_r_* = 867.97	*F*(000) = 852
Triclinic, *P*1	*D*_x_ = 2.099 Mg m^−^^3^
Hall symbol: -P 1	Mo *K*α radiation, λ = 0.71073 Å
*a* = 7.7774 (9) Å	Cell parameters from 16661 reflections
*b* = 10.2465 (12) Å	θ = 2.5–27.5°
*c* = 17.773 (2) Å	µ = 2.06 mm^−^^1^
α = 80.255 (5)°	*T* = 150 K
β = 81.760 (5)°	Block, colourless
γ = 82.547 (5)°	0.32 × 0.32 × 0.13 mm
*V* = 1373.4 (3) Å^3^	

### Data collection

**Table:**

Bruker SMART 1000 CCD diffractometer	6323 independent reflections
Radiation source: fine-focus sealed tube	5762 reflections with *I* > 2σ(*I*)
graphite	*R*_int_ = 0.027
φ and ω scans	θ_max_ = 27.6°, θ_min_ = 1.2°
Absorption correction: multi-scan (*SADABS*; Bruker, 2001)	*h* = −10→10
*T*_min_ = 0.559, *T*_max_ = 0.776	*k* = −13→13
29846 measured reflections	*l* = −23→23

### Refinement

**Table:**

Refinement on *F*^2^	Primary atom site location: structure-invariant direct methods
Least-squares matrix: full	Secondary atom site location: difference Fourier map
*R*[*F*^2^ > 2σ(*F*^2^)] = 0.021	Hydrogen site location: inferred from neighbouring sites
*wR*(*F*^2^) = 0.054	H-atom parameters constrained
*S* = 1.05	*w* = 1/[σ^2^(*F*_o_^2^) + (0.0291*P*)^2^ + 1.1343*P*] where *P* = (*F*_o_^2^ + 2*F*_c_^2^)/3
6323 reflections	(Δ/σ)_max_ = 0.002
415 parameters	Δρ_max_ = 1.02 e Å^−^^3^
0 restraints	Δρ_min_ = −0.61 e Å^−^^3^

### Fractional atomic coordinates and isotropic or equivalent isotropic displacement parameters (Å^2^)

**Table:**

	*x*	*y*	*z*	*U*_iso_*/*U*_eq_	
Sb1	0.580591 (16)	0.703688 (13)	0.358013 (7)	0.01090 (4)	
Sb2	0.438680 (16)	0.752017 (12)	0.129483 (7)	0.01011 (4)	
O1W	0.11728 (19)	0.69329 (15)	0.33900 (9)	0.0163 (3)	
H1A	0.1420	0.6243	0.3712	0.020*	
H1B	0.0115	0.6949	0.3314	0.020*	
O1	0.72456 (19)	0.67130 (15)	0.45745 (8)	0.0150 (3)	
O2W	0.9042 (2)	0.8814 (2)	0.17638 (12)	0.0439 (6)	
H2A	0.8878	0.9460	0.1396	0.053*	
H2B	1.0123	0.8520	0.1710	0.053*	
O2	0.8151 (2)	0.52864 (15)	0.55669 (9)	0.0170 (3)	
O3	0.7165 (2)	0.08698 (15)	0.46954 (9)	0.0189 (3)	
O4	0.4341 (2)	0.39130 (15)	0.24797 (9)	0.0172 (3)	
O5	0.49471 (19)	0.58946 (14)	0.26877 (8)	0.0141 (3)	
O6	0.30385 (19)	0.77857 (14)	0.02920 (8)	0.0145 (3)	
O7	0.1851 (2)	0.91893 (15)	−0.06395 (9)	0.0186 (3)	
O8	0.23816 (19)	1.36547 (14)	0.03077 (8)	0.0144 (3)	
O9	0.5309 (2)	1.06860 (15)	0.25055 (9)	0.0221 (4)	
O10	0.49467 (19)	0.86694 (14)	0.22539 (8)	0.0137 (3)	
O11	0.80104 (18)	0.69280 (15)	0.29264 (8)	0.0155 (3)	
H11A	0.7985	0.7579	0.2560	0.019*	
O13	0.6824 (3)	0.92019 (17)	0.36682 (10)	0.0296 (4)	
H13B	0.6856	0.9689	0.4007	0.035*	
H13A	0.6356	0.9734	0.3315	0.035*	
O15	0.4245 (2)	0.51911 (15)	0.10194 (9)	0.0172 (3)	
H15B	0.3615	0.4804	0.0792	0.021*	
H15A	0.4127	0.4729	0.1465	0.021*	
O16	0.21023 (18)	0.73191 (15)	0.18620 (8)	0.0146 (3)	
H16A	0.2120	0.7088	0.2346	0.018*	
N1	0.6307 (2)	0.48763 (17)	0.39380 (10)	0.0111 (3)	
N2	0.3608 (2)	0.96575 (17)	0.09960 (9)	0.0109 (3)	
N3	−0.1645 (2)	0.95121 (18)	−0.40617 (10)	0.0148 (4)	
H3A	−0.2043	1.0024	−0.4440	0.018*	
N4	0.1423 (2)	0.50965 (18)	−0.09767 (10)	0.0141 (3)	
H4A	0.1802	0.4608	−0.0586	0.017*	
C1	0.7559 (3)	0.5527 (2)	0.49466 (12)	0.0128 (4)	
C2	0.7105 (3)	0.4426 (2)	0.45717 (11)	0.0111 (4)	
C3	0.7429 (3)	0.3094 (2)	0.48443 (12)	0.0128 (4)	
H3	0.7997	0.2821	0.5279	0.015*	
C4	0.6890 (3)	0.2126 (2)	0.44593 (11)	0.0131 (4)	
C5	0.6039 (3)	0.2645 (2)	0.37913 (12)	0.0132 (4)	
H5	0.5654	0.2068	0.3516	0.016*	
C6	0.5793 (3)	0.3989 (2)	0.35577 (11)	0.0113 (4)	
C7	0.4945 (3)	0.4624 (2)	0.28500 (11)	0.0117 (4)	
C8	0.2517 (3)	0.8969 (2)	−0.00416 (12)	0.0128 (4)	
C9	0.2806 (3)	1.0087 (2)	0.03616 (11)	0.0116 (4)	
C10	0.2339 (3)	1.1418 (2)	0.01168 (11)	0.0117 (4)	
H10	0.1768	1.1676	−0.0319	0.014*	
C11	0.2727 (3)	1.2403 (2)	0.05286 (11)	0.0114 (4)	
C12	0.3553 (3)	1.1902 (2)	0.12072 (12)	0.0129 (4)	
H12	0.3818	1.2491	0.1508	0.016*	
C13	0.3953 (3)	1.0562 (2)	0.14123 (11)	0.0117 (4)	
C14	0.4814 (3)	0.9955 (2)	0.21167 (12)	0.0133 (4)	
C15	0.1954 (3)	0.6317 (2)	−0.11368 (12)	0.0146 (4)	
H15	0.2685	0.6569	−0.0829	0.017*	
C16	0.1420 (3)	0.7203 (2)	−0.17571 (12)	0.0142 (4)	
H16	0.1806	0.8044	−0.1873	0.017*	
C17	0.0292 (3)	0.6824 (2)	−0.22106 (11)	0.0118 (4)	
C18	−0.0372 (3)	0.7772 (2)	−0.28624 (11)	0.0118 (4)	
C19	−0.0412 (3)	0.9149 (2)	−0.28961 (12)	0.0155 (4)	
H19	−0.0004	0.9490	−0.2510	0.019*	
C20	−0.1056 (3)	1.0000 (2)	−0.35033 (12)	0.0170 (4)	
H20	−0.1082	1.0916	−0.3526	0.020*	
C21	−0.1612 (3)	0.8204 (2)	−0.40566 (12)	0.0153 (4)	
H21	−0.2014	0.7896	−0.4455	0.018*	
C22	−0.0982 (3)	0.7308 (2)	−0.34594 (12)	0.0139 (4)	
H22	−0.0964	0.6397	−0.3455	0.017*	
C23	−0.0233 (3)	0.5547 (2)	−0.20217 (12)	0.0148 (4)	
H23	−0.0977	0.5270	−0.2314	0.018*	
C24	0.0355 (3)	0.4695 (2)	−0.14003 (12)	0.0154 (4)	
H24	0.0010	0.3841	−0.1275	0.019*	

### Atomic displacement parameters (Å^2^)

**Table:**

	*U*^11^	*U*^22^	*U*^33^	*U*^12^	*U*^13^	*U*^23^
Sb1	0.01408 (7)	0.00955 (7)	0.00914 (7)	−0.00086 (5)	−0.00371 (5)	−0.00003 (5)
Sb2	0.01203 (7)	0.00898 (7)	0.00915 (7)	−0.00093 (5)	−0.00270 (5)	0.00010 (5)
O1W	0.0153 (7)	0.0179 (8)	0.0148 (7)	−0.0018 (6)	−0.0057 (6)	0.0032 (6)
O1	0.0225 (8)	0.0107 (7)	0.0125 (7)	−0.0030 (6)	−0.0072 (6)	0.0011 (6)
O2W	0.0242 (9)	0.0498 (13)	0.0392 (12)	0.0090 (9)	0.0027 (8)	0.0303 (10)
O2	0.0273 (8)	0.0135 (7)	0.0117 (7)	−0.0020 (6)	−0.0095 (6)	−0.0005 (6)
O3	0.0331 (9)	0.0092 (7)	0.0156 (7)	−0.0017 (6)	−0.0117 (6)	0.0016 (6)
O4	0.0273 (8)	0.0122 (7)	0.0145 (7)	−0.0043 (6)	−0.0110 (6)	0.0000 (6)
O5	0.0191 (7)	0.0102 (7)	0.0139 (7)	−0.0020 (6)	−0.0069 (6)	0.0003 (6)
O6	0.0214 (7)	0.0097 (7)	0.0134 (7)	−0.0017 (6)	−0.0070 (6)	−0.0001 (6)
O7	0.0302 (8)	0.0138 (7)	0.0139 (7)	−0.0005 (6)	−0.0122 (6)	−0.0013 (6)
O8	0.0220 (7)	0.0084 (7)	0.0128 (7)	−0.0002 (6)	−0.0066 (6)	0.0011 (5)
O9	0.0396 (10)	0.0115 (7)	0.0190 (8)	−0.0024 (7)	−0.0175 (7)	−0.0012 (6)
O10	0.0197 (7)	0.0086 (7)	0.0135 (7)	−0.0017 (6)	−0.0063 (6)	0.0001 (6)
O11	0.0145 (7)	0.0172 (8)	0.0131 (7)	−0.0020 (6)	−0.0030 (6)	0.0035 (6)
O13	0.0574 (12)	0.0132 (8)	0.0231 (9)	−0.0055 (8)	−0.0228 (8)	−0.0006 (7)
O15	0.0258 (8)	0.0110 (7)	0.0162 (7)	−0.0043 (6)	−0.0087 (6)	0.0006 (6)
O16	0.0139 (7)	0.0180 (8)	0.0112 (7)	−0.0031 (6)	−0.0021 (5)	0.0012 (6)
N1	0.0140 (8)	0.0104 (8)	0.0089 (8)	−0.0012 (6)	−0.0033 (6)	0.0001 (6)
N2	0.0131 (8)	0.0098 (8)	0.0096 (8)	−0.0008 (6)	−0.0028 (6)	0.0000 (6)
N3	0.0184 (8)	0.0148 (9)	0.0105 (8)	−0.0016 (7)	−0.0050 (7)	0.0030 (7)
N4	0.0177 (8)	0.0129 (8)	0.0106 (8)	0.0020 (7)	−0.0042 (7)	0.0003 (7)
C1	0.0151 (9)	0.0120 (9)	0.0110 (9)	−0.0006 (7)	−0.0016 (7)	−0.0018 (8)
C2	0.0124 (9)	0.0135 (10)	0.0079 (9)	−0.0028 (7)	−0.0020 (7)	−0.0012 (7)
C3	0.0160 (9)	0.0126 (10)	0.0094 (9)	−0.0013 (7)	−0.0041 (7)	0.0015 (7)
C4	0.0170 (9)	0.0115 (9)	0.0103 (9)	−0.0012 (8)	−0.0024 (7)	0.0000 (7)
C5	0.0161 (9)	0.0129 (10)	0.0113 (9)	−0.0022 (7)	−0.0034 (7)	−0.0014 (8)
C6	0.0122 (9)	0.0127 (9)	0.0090 (9)	−0.0013 (7)	−0.0025 (7)	−0.0011 (7)
C7	0.0129 (9)	0.0110 (9)	0.0100 (9)	0.0005 (7)	−0.0022 (7)	0.0010 (7)
C8	0.0155 (9)	0.0111 (9)	0.0114 (9)	−0.0024 (7)	−0.0016 (7)	−0.0003 (8)
C9	0.0127 (9)	0.0123 (10)	0.0097 (9)	−0.0014 (7)	−0.0012 (7)	−0.0012 (7)
C10	0.0130 (9)	0.0130 (10)	0.0090 (9)	−0.0018 (7)	−0.0038 (7)	0.0005 (7)
C11	0.0132 (9)	0.0104 (9)	0.0095 (9)	−0.0004 (7)	−0.0002 (7)	0.0002 (7)
C12	0.0161 (9)	0.0112 (10)	0.0113 (9)	−0.0014 (7)	−0.0032 (7)	−0.0003 (8)
C13	0.0137 (9)	0.0114 (9)	0.0099 (9)	−0.0011 (7)	−0.0023 (7)	−0.0010 (7)
C14	0.0167 (9)	0.0117 (10)	0.0111 (9)	−0.0010 (7)	−0.0041 (7)	0.0005 (8)
C15	0.0153 (9)	0.0144 (10)	0.0157 (10)	−0.0015 (8)	−0.0054 (8)	−0.0045 (8)
C16	0.0166 (9)	0.0115 (10)	0.0153 (10)	−0.0033 (8)	−0.0035 (8)	−0.0012 (8)
C17	0.0121 (9)	0.0118 (9)	0.0105 (9)	0.0002 (7)	0.0001 (7)	−0.0016 (8)
C18	0.0121 (9)	0.0115 (9)	0.0112 (9)	−0.0021 (7)	−0.0017 (7)	0.0007 (8)
C19	0.0215 (10)	0.0133 (10)	0.0129 (10)	−0.0029 (8)	−0.0054 (8)	−0.0019 (8)
C20	0.0224 (10)	0.0133 (10)	0.0158 (10)	−0.0026 (8)	−0.0053 (8)	−0.0007 (8)
C21	0.0179 (10)	0.0169 (10)	0.0120 (10)	−0.0035 (8)	−0.0051 (8)	−0.0010 (8)
C22	0.0177 (10)	0.0118 (10)	0.0128 (10)	−0.0031 (8)	−0.0040 (8)	−0.0011 (8)
C23	0.0171 (10)	0.0147 (10)	0.0140 (10)	−0.0035 (8)	−0.0061 (8)	−0.0012 (8)
C24	0.0195 (10)	0.0120 (10)	0.0152 (10)	−0.0032 (8)	−0.0048 (8)	0.0004 (8)

### Geometric parameters (Å, °)

**Table:**

Sb1—O11	1.9300 (15)	N3—H3A	0.8500
Sb1—O1	2.1816 (14)	N4—C15	1.340 (3)
Sb1—N1	2.1972 (17)	N4—C24	1.341 (3)
Sb1—O5	2.3381 (15)	N4—H4A	0.8500
Sb1—O13	2.4861 (17)	C1—C2	1.505 (3)
Sb1—O10	2.7617 (14)	C2—C3	1.372 (3)
Sb2—O16	1.9304 (14)	C3—C4	1.431 (3)
Sb2—O6	2.1559 (14)	C3—H3	0.9300
Sb2—N2	2.1908 (17)	C4—C5	1.432 (3)
Sb2—O10	2.3468 (15)	C5—C6	1.366 (3)
Sb2—O15	2.5350 (15)	C5—H5	0.9300
Sb2—O5	2.7959 (14)	C6—C7	1.513 (3)
O1W—H1A	0.8500	C8—C9	1.506 (3)
O1W—H1B	0.8500	C9—C10	1.378 (3)
O1—C1	1.293 (2)	C10—C11	1.428 (3)
O2W—H2A	0.8589	C10—H10	0.9300
O2W—H2B	0.8529	C11—C12	1.435 (3)
O2—C1	1.230 (2)	C12—C13	1.366 (3)
O3—C4	1.284 (3)	C12—H12	0.9300
O4—C7	1.235 (3)	C13—C14	1.507 (3)
O5—C7	1.285 (3)	C15—C16	1.380 (3)
O6—C8	1.297 (2)	C15—H15	0.9300
O7—C8	1.223 (2)	C16—C17	1.402 (3)
O8—C11	1.281 (2)	C16—H16	0.9300
O9—C14	1.230 (3)	C17—C23	1.393 (3)
O10—C14	1.291 (3)	C17—C18	1.487 (3)
O11—H11A	0.8500	C18—C22	1.397 (3)
O13—H13B	0.8499	C18—C19	1.399 (3)
O13—H13A	0.8500	C19—C20	1.378 (3)
O15—H15B	0.8500	C19—H19	0.9300
O15—H15A	0.8501	C20—H20	0.9300
O16—H16A	0.8549	C21—C22	1.383 (3)
N1—C2	1.348 (2)	C21—H21	0.9300
N1—C6	1.350 (3)	C22—H22	0.9300
N2—C9	1.347 (3)	C23—C24	1.381 (3)
N2—C13	1.353 (3)	C23—H23	0.9300
N3—C21	1.336 (3)	C24—H24	0.9300
N3—C20	1.342 (3)		
			
O11—Sb1—O1	88.73 (6)	C2—C3—C4	120.02 (18)
O11—Sb1—N1	87.40 (6)	C2—C3—H3	120.0
O1—Sb1—N1	72.28 (6)	C4—C3—H3	120.0
O11—Sb1—O5	82.88 (6)	O3—C4—C3	122.05 (18)
O1—Sb1—O5	141.44 (5)	O3—C4—C5	122.18 (19)
N1—Sb1—O5	69.80 (6)	C3—C4—C5	115.77 (18)
O11—Sb1—O13	79.47 (7)	C6—C5—C4	120.04 (19)
O1—Sb1—O13	72.89 (5)	C6—C5—H5	120.0
N1—Sb1—O13	142.91 (6)	C4—C5—H5	120.0
O5—Sb1—O13	140.84 (5)	N1—C6—C5	122.63 (18)
O11—Sb1—O10	78.80 (5)	N1—C6—C7	113.70 (17)
O1—Sb1—O10	148.04 (5)	C5—C6—C7	123.67 (18)
N1—Sb1—O10	135.33 (5)	O4—C7—O5	125.85 (18)
O5—Sb1—O10	66.46 (5)	O4—C7—C6	119.32 (18)
O13—Sb1—O10	75.93 (5)	O5—C7—C6	114.82 (17)
O16—Sb2—O6	84.67 (6)	O7—C8—O6	123.94 (19)
O16—Sb2—N2	89.44 (6)	O7—C8—C9	121.27 (18)
O6—Sb2—N2	72.66 (6)	O6—C8—C9	114.78 (17)
O16—Sb2—O10	87.61 (6)	N2—C9—C10	122.26 (19)
O6—Sb2—O10	141.57 (5)	N2—C9—C8	112.91 (17)
N2—Sb2—O10	69.67 (6)	C10—C9—C8	124.82 (18)
O16—Sb2—O15	84.54 (6)	C9—C10—C11	120.29 (18)
O6—Sb2—O15	74.52 (5)	C9—C10—H10	119.9
N2—Sb2—O15	147.04 (6)	C11—C10—H10	119.9
O10—Sb2—O15	142.00 (5)	O8—C11—C10	122.67 (18)
O16—Sb2—O5	73.88 (5)	O8—C11—C12	121.74 (18)
O6—Sb2—O5	145.16 (5)	C10—C11—C12	115.58 (18)
N2—Sb2—O5	132.68 (5)	C13—C12—C11	120.15 (19)
O10—Sb2—O5	65.76 (5)	C13—C12—H12	119.9
O15—Sb2—O5	76.35 (5)	C11—C12—H12	119.9
H1A—O1W—H1B	108.1	N2—C13—C12	122.65 (18)
C1—O1—Sb1	120.34 (13)	N2—C13—C14	113.87 (17)
H2A—O2W—H2B	107.6	C12—C13—C14	123.48 (18)
C7—O5—Sb1	118.75 (12)	O9—C14—O10	125.98 (19)
C7—O5—Sb2	127.75 (12)	O9—C14—C13	119.46 (18)
Sb1—O5—Sb2	113.14 (6)	O10—C14—C13	114.56 (17)
C8—O6—Sb2	120.80 (13)	N4—C15—C16	120.29 (19)
C14—O10—Sb2	118.36 (12)	N4—C15—H15	119.9
C14—O10—Sb1	127.57 (12)	C16—C15—H15	119.9
Sb2—O10—Sb1	114.08 (5)	C15—C16—C17	119.5 (2)
Sb1—O11—H11A	108.5	C15—C16—H16	120.2
Sb1—O13—H13B	138.7	C17—C16—H16	120.2
Sb1—O13—H13A	103.0	C23—C17—C16	118.27 (18)
H13B—O13—H13A	103.5	C23—C17—C18	120.70 (19)
Sb2—O15—H15B	137.7	C16—C17—C18	121.00 (19)
Sb2—O15—H15A	103.5	C22—C18—C19	118.02 (18)
H15B—O15—H15A	99.8	C22—C18—C17	120.69 (19)
Sb2—O16—H16A	113.5	C19—C18—C17	121.29 (19)
C2—N1—C6	119.08 (17)	C20—C19—C18	119.8 (2)
C2—N1—Sb1	118.77 (13)	C20—C19—H19	120.1
C6—N1—Sb1	122.11 (13)	C18—C19—H19	120.1
C9—N2—C13	119.02 (17)	N3—C20—C19	120.3 (2)
C9—N2—Sb2	118.66 (13)	N3—C20—H20	119.9
C13—N2—Sb2	122.29 (13)	C19—C20—H20	119.9
C21—N3—C20	121.90 (18)	N3—C21—C22	120.11 (19)
C21—N3—H3A	116.7	N3—C21—H21	119.9
C20—N3—H3A	121.4	C22—C21—H21	119.9
C15—N4—C24	121.93 (18)	C21—C22—C18	119.90 (19)
C15—N4—H4A	115.0	C21—C22—H22	120.0
C24—N4—H4A	123.1	C18—C22—H22	120.0
O2—C1—O1	123.94 (19)	C24—C23—C17	119.90 (19)
O2—C1—C2	121.16 (19)	C24—C23—H23	120.0
O1—C1—C2	114.88 (17)	C17—C23—H23	120.0
N1—C2—C3	122.44 (19)	N4—C24—C23	120.1 (2)
N1—C2—C1	113.16 (17)	N4—C24—H24	120.0
C3—C2—C1	124.39 (18)	C23—C24—H24	120.0
			
O11—Sb1—O1—C1	−94.49 (15)	Sb1—N1—C2—C1	−0.4 (2)
N1—Sb1—O1—C1	−6.82 (14)	O2—C1—C2—N1	173.49 (19)
O5—Sb1—O1—C1	−17.6 (2)	O1—C1—C2—N1	−5.3 (3)
O13—Sb1—O1—C1	−173.86 (16)	O2—C1—C2—C3	−5.3 (3)
O10—Sb1—O1—C1	−160.75 (13)	O1—C1—C2—C3	175.91 (19)
O11—Sb1—O5—C7	98.21 (15)	N1—C2—C3—C4	−0.9 (3)
O1—Sb1—O5—C7	19.31 (19)	C1—C2—C3—C4	177.82 (19)
N1—Sb1—O5—C7	8.37 (14)	C2—C3—C4—O3	−179.9 (2)
O13—Sb1—O5—C7	161.79 (14)	C2—C3—C4—C5	0.5 (3)
O10—Sb1—O5—C7	179.06 (16)	O3—C4—C5—C6	−179.3 (2)
O11—Sb1—O5—Sb2	−75.41 (7)	C3—C4—C5—C6	0.2 (3)
O1—Sb1—O5—Sb2	−154.31 (7)	C2—N1—C6—C5	0.4 (3)
N1—Sb1—O5—Sb2	−165.25 (8)	Sb1—N1—C6—C5	−177.52 (15)
O13—Sb1—O5—Sb2	−11.83 (12)	C2—N1—C6—C7	−178.94 (17)
O10—Sb1—O5—Sb2	5.44 (4)	Sb1—N1—C6—C7	3.1 (2)
O16—Sb2—O5—C7	85.91 (16)	C4—C5—C6—N1	−0.7 (3)
O6—Sb2—O5—C7	31.7 (2)	C4—C5—C6—C7	178.54 (18)
N2—Sb2—O5—C7	159.63 (15)	Sb1—O5—C7—O4	171.21 (16)
O10—Sb2—O5—C7	−179.36 (17)	Sb2—O5—C7—O4	−16.2 (3)
O15—Sb2—O5—C7	−2.28 (15)	Sb1—O5—C7—C6	−9.6 (2)
O16—Sb2—O5—Sb1	−101.17 (7)	Sb2—O5—C7—C6	163.02 (12)
O6—Sb2—O5—Sb1	−155.40 (7)	N1—C6—C7—O4	−176.23 (18)
N2—Sb2—O5—Sb1	−27.44 (10)	C5—C6—C7—O4	4.4 (3)
O10—Sb2—O5—Sb1	−6.43 (5)	N1—C6—C7—O5	4.5 (3)
O15—Sb2—O5—Sb1	170.65 (7)	C5—C6—C7—O5	−174.85 (19)
O16—Sb2—O6—C8	95.22 (15)	Sb2—O6—C8—O7	174.97 (16)
N2—Sb2—O6—C8	4.14 (15)	Sb2—O6—C8—C9	−4.5 (2)
O10—Sb2—O6—C8	15.9 (2)	C13—N2—C9—C10	0.6 (3)
O15—Sb2—O6—C8	−178.98 (16)	Sb2—N2—C9—C10	−177.32 (15)
O5—Sb2—O6—C8	146.75 (13)	C13—N2—C9—C8	179.79 (17)
O16—Sb2—O10—C14	−100.59 (15)	Sb2—N2—C9—C8	1.8 (2)
O6—Sb2—O10—C14	−22.27 (19)	O7—C8—C9—N2	−177.86 (19)
N2—Sb2—O10—C14	−10.30 (14)	O6—C8—C9—N2	1.6 (3)
O15—Sb2—O10—C14	−178.59 (13)	O7—C8—C9—C10	1.3 (3)
O5—Sb2—O10—C14	−173.98 (16)	O6—C8—C9—C10	−179.23 (19)
O16—Sb2—O10—Sb1	78.87 (7)	N2—C9—C10—C11	1.2 (3)
O6—Sb2—O10—Sb1	157.19 (7)	C8—C9—C10—C11	−177.87 (18)
N2—Sb2—O10—Sb1	169.16 (8)	C9—C10—C11—O8	177.21 (19)
O15—Sb2—O10—Sb1	0.88 (11)	C9—C10—C11—C12	−2.1 (3)
O5—Sb2—O10—Sb1	5.48 (5)	O8—C11—C12—C13	−177.93 (19)
O11—Sb1—O10—C14	−100.08 (17)	C10—C11—C12—C13	1.4 (3)
O1—Sb1—O10—C14	−31.2 (2)	C9—N2—C13—C12	−1.4 (3)
N1—Sb1—O10—C14	−174.64 (15)	Sb2—N2—C13—C12	176.48 (15)
O5—Sb1—O10—C14	172.88 (17)	C9—N2—C13—C14	178.68 (17)
O13—Sb1—O10—C14	−18.26 (16)	Sb2—N2—C13—C14	−3.4 (2)
O11—Sb1—O10—Sb2	80.52 (7)	C11—C12—C13—N2	0.3 (3)
O1—Sb1—O10—Sb2	149.42 (8)	C11—C12—C13—C14	−179.77 (18)
N1—Sb1—O10—Sb2	5.96 (11)	Sb2—O10—C14—O9	−168.47 (18)
O5—Sb1—O10—Sb2	−6.53 (5)	Sb1—O10—C14—O9	12.2 (3)
O13—Sb1—O10—Sb2	162.33 (8)	Sb2—O10—C14—C13	12.0 (2)
O11—Sb1—N1—C2	92.96 (15)	Sb1—O10—C14—C13	−167.43 (12)
O1—Sb1—N1—C2	3.46 (14)	N2—C13—C14—O9	174.43 (19)
O5—Sb1—N1—C2	176.33 (16)	C12—C13—C14—O9	−5.5 (3)
O13—Sb1—N1—C2	24.3 (2)	N2—C13—C14—O10	−6.0 (3)
O10—Sb1—N1—C2	164.14 (12)	C12—C13—C14—O10	174.13 (19)
O11—Sb1—N1—C6	−89.12 (16)	C24—N4—C15—C16	−0.7 (3)
O1—Sb1—N1—C6	−178.62 (17)	N4—C15—C16—C17	1.2 (3)
O5—Sb1—N1—C6	−5.75 (14)	C15—C16—C17—C23	−0.9 (3)
O13—Sb1—N1—C6	−157.80 (14)	C15—C16—C17—C18	177.29 (19)
O10—Sb1—N1—C6	−17.93 (19)	C23—C17—C18—C22	−23.5 (3)
O16—Sb2—N2—C9	−87.61 (15)	C16—C17—C18—C22	158.33 (19)
O6—Sb2—N2—C9	−3.03 (14)	C23—C17—C18—C19	156.3 (2)
O10—Sb2—N2—C9	−175.27 (16)	C16—C17—C18—C19	−21.9 (3)
O15—Sb2—N2—C9	−8.6 (2)	C22—C18—C19—C20	0.6 (3)
O5—Sb2—N2—C9	−154.87 (13)	C17—C18—C19—C20	−179.2 (2)
O16—Sb2—N2—C13	94.50 (16)	C21—N3—C20—C19	−0.8 (3)
O6—Sb2—N2—C13	179.08 (17)	C18—C19—C20—N3	0.0 (3)
O10—Sb2—N2—C13	6.84 (14)	C20—N3—C21—C22	0.9 (3)
O15—Sb2—N2—C13	173.56 (13)	N3—C21—C22—C18	−0.2 (3)
O5—Sb2—N2—C13	27.24 (18)	C19—C18—C22—C21	−0.5 (3)
Sb1—O1—C1—O2	−169.95 (16)	C17—C18—C22—C21	179.29 (19)
Sb1—O1—C1—C2	8.8 (2)	C16—C17—C23—C24	0.2 (3)
C6—N1—C2—C3	0.4 (3)	C18—C17—C23—C24	−178.03 (19)
Sb1—N1—C2—C3	178.41 (15)	C15—N4—C24—C23	−0.1 (3)
C6—N1—C2—C1	−178.42 (17)	C17—C23—C24—N4	0.3 (3)

### Hydrogen-bond geometry (Å, °)

**Table:**

*D*—H···*A*	*D*—H	H···*A*	*D*···*A*	*D*—H···*A*
O1W—H1A···O2^i^	0.85	1.88	2.725 (2)	177
O1W—H1B···O11^ii^	0.85	1.87	2.704 (2)	167
O2W—H2A···O7^iii^	0.86	1.85	2.703 (2)	171
O2W—H2B···O16^iv^	0.85	1.86	2.669 (2)	157
O11—H11A···O2W	0.85	1.90	2.688 (2)	154
O16—H16A···O1W	0.85	1.88	2.684 (2)	155
O13—H13A···O9	0.85	1.83	2.672 (2)	173
O13—H13B···O3^v^	0.85	1.92	2.764 (2)	173
O15—H15A···O4	0.85	1.88	2.709 (2)	166
O15—H15B···O8^vi^	0.85	1.98	2.823 (2)	171
N3—H3A···O3^vii^	0.85	1.78	2.624 (2)	171
N4—H4A···O8^vi^	0.85	1.80	2.648 (2)	173
C3—H3···O1W^i^	0.93	2.60	3.460 (3)	154
C5—H5···O9^vi^	0.93	2.53	3.434 (3)	164
C12—H12···O4^v^	0.93	2.55	3.470 (3)	172
C15—H15···O6	0.93	2.59	3.415 (3)	149
C19—H19···O2W^iii^	0.93	2.60	3.484 (3)	160
C20—H20···O1W^viii^	0.93	2.25	3.172 (3)	172
C21—H21···O1^ix^	0.93	2.44	3.358 (3)	170
C22—H22···O2^ix^	0.93	2.45	3.107 (3)	128
C24—H24···O16^x^	0.93	2.57	3.261 (3)	131

Symmetry codes: (i) −*x*+1, −*y*+1, −*z*+1; (ii) *x*−1, *y*, *z*; (iii) −*x*+1, −*y*+2, −*z*; (iv) *x*+1, *y*, *z*; (v) *x*, *y*+1, *z*; (vi) *x*, *y*−1, *z*; (vii) *x*−1, *y*+1, *z*−1; (viii) −*x*, −*y*+2, −*z*; (ix) *x*−1, *y*, *z*−1; (x) −*x*, −*y*+1, −*z*.
